# Improving cataract output in India: a joined-up approach

**Published:** 2022-12-16

**Authors:** Madhu Bhadauria, Anshu Singh

**Affiliations:** 1 Chief Medical Officer: Sitapur Eye Hospital, Sitapur, India.; 2 Program Manager: Sitapur Eye Hospital, Sitapur, India.

**Improvements in outreach, demand generation, training, and quality control yielded a 15-fold increase in cataract output in just over a decade at Sitapur Eye Hospital.**

**Figure:**
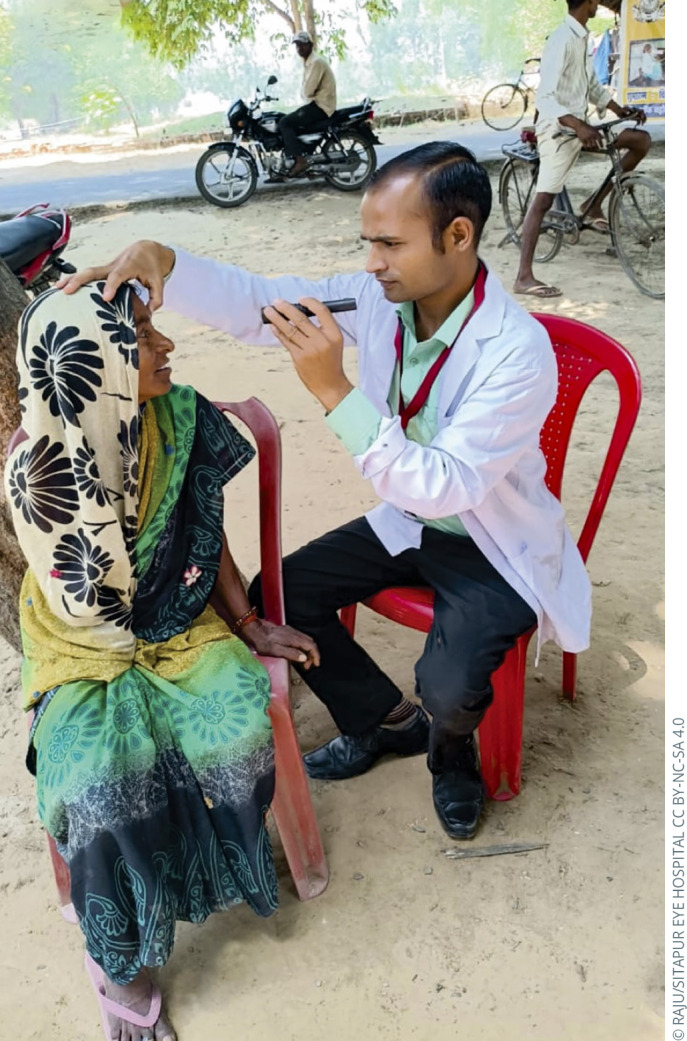
Outreach services improve access to eye care for women, children, and disabled people. **INDIA**

Sitapur Eye Hospital was set up in 1927 in Sitapur, in the Indian state of Uttar Pradesh. The hospital began cataract services in 1935,^1^ providing much-needed community eye care for many decades. However, there followed a period of decline in eye care services, with the hospital’s cataract output reducing to just 2,000 operations per year in 2009. The hospital’s extensive buildings also degraded over time.

The prevalence of cataract in Uttar Pradesh is high because of poor access to surgery.^2^ High quality, high-volume eye care centres are therefore needed, and Sitapur Eye Hospital (SEH), with its large physical infrastructure and recognisable brand value, had the potential to increase its cataract output to better meet the eye care needs of the population.

A team was formed in 2009 to do just that. They found that the key challenges were as follows:

- a shortage of eye care personnel with the right level of skills in the right areas
- a lack of systems to monitor and improve quality
- not enough patients coming in for surgery
- a lack of proper counselling for patients needing surgery, and a lack of effective tracking of patients, e.g., by sending follow-up reminders
- a lack of outreach activities
- a shortage of funds
- staff attitudes that compromised patient care.

Over the next 13 years, these challenges were addressed through improvements in the following areas.

## Systems, infrastructure, training, and quality

Improvements have included the installation of better equipment, training of ophthalmic personnel, renovation of the operation theatre, and putting in place better systems flow and processes. Information and hospital management system (IHMS) software was installed to electronically record the demographic and clinic data of patients. Outpatient processes were streamlined. All clinical and administrative protocols were also aligned to the country’s standard operating procedures; these were strictly followed and monitored to avoid any medical errors, such as cluster endophthalmitis. The visual outcomes of cataract surgery were assessed using Cataract Quality Metrics, a benchmarking software programme. Infrastructure and quality improvement is a continual process now, which is built into our organisational culture.

The academic/training programmes that we now offer includes a Bachelor of Optometry degree, a Masters in Ophthalmology degree (with an annual intake of 15 students), clinical fellowships in ophthalmology and optometry, and training courses for ophthalmologists, optometrists, and ophthalmic assistants.

## Collaborations

Organisations such as Aravind Eye Care System, Sightsavers India, CBM, and Orbis, as well as the Indian government – through health schemes like the national programme for control of blindness and visual impairment, ‘Rashtriya Bal Swasthya Karyakram’ (a national programme to protect and promote the health of children) – are collaborating with us to improve service quality, offer training, and improve service delivery; they are also providing financial support.

## Increasing patients’ access to surgery

Sitapur Eye Hospital conducts comprehensive eye care outreach camps where we identify cataract patients in rural and low-income communities. Everyone selected for cataract surgery at the camps is offered free eye surgery, spectacles, medicines, transport, and food. During the COVID-19 pandemic, door-to-door screening and mobile van-based services were created to reach the community. For non-surgical eye conditions, twenty well-equipped vision centres have also been established in eight districts; these enable communities to have easier access to eye care in their local neighbourhoods.

## Finances

Initially, Sitapur Eye Hospital’s chief medical officer, who is responsible for teaching and administration, helped to generate income by performing phacoemulsification using premium lenses, glaucoma surgery, and paediatric surgery. This subsidised the cost of providing care to patients who would otherwise be unable to afford surgery.

Finances now come from multiple sources. Sitapur Eye Hospital has set up a three-tier paying system for patients: paid services for those who can afford to pay in full, subsidised services for those unable to pay the full fees, and free treatment for those unable to pay at all. The ratio of paying to free patients is 30:70. The hospital also receives funding from non-governmental organisations (which support their special outreach activities), via government reimbursements and medical insurance, and from the sale of spectacles and medicines. All of this has enabled us to become financially self-sustaining.

## Results

As a result of these efforts, Sitapur Eye Hospital has increased its output from 2,000 cataract operations in 2009 to 31,000 operations in 2021, with no episodes of cluster endophthalmitis and 74.8% of patients achieving corrected visual acuity of 6/18 or better. We operate on 700 to 800 children for cataract annually and attend to all sub-specialty cases. According to the demographic data collected using our IHMS, the male-to-female ratio of cataract patients is 50:50. Because of our outreach services, eye care is now reaching more villages, which is improving access to services for women, children, and disabled people, on their doorstep.

## Looking to the future

The Sitapur Eye Hospital model is a self-sustainable one, both financially and in terms of human resource needs. Infrastructure and quality improvement is now a continual process, built into our organisational culture. The aim is to perform 100,000 cataract operations annually by 2030, to continue to provide equitable eye care for all, to develop specialties in ophthalmology, and to upgrade training and research facilities on an ongoing basis.
